# Clinical impact and misdiagnosis of functional ophthalmological symptoms: a case report

**DOI:** 10.1186/s13256-023-04063-0

**Published:** 2023-08-11

**Authors:** Beatriz Pozuelo Moyano, Catherine Duquenne, Bernard Favrat, Borruat Francois-Xavier, Ioannis Kokkinakis, Konstantinos Tzartzas

**Affiliations:** 1 Center for Primary Care and Public Health (Unisanté), University of Lausanne, Rue du Bugnon 44, 1011 Lausanne, Switzerland; 2 Neuro-Ophtalmology Unit, Hospital of Jules-Gonin, Lausanne, Switzerland

**Keywords:** Somatoform disorder, Factitious disorder, Disability, Functional blindness

## Abstract

**Background:**

There is a high prevalence of somatoform disorders and medically unexplained symptoms. When it comes to deciding whether a patient is able to work, it is essential to differentiate a somatoform disorder from a factitious disorder. The case presented demonstrates the impact on disability benefits and the subsequent psychosocial repercussions of misdiagnosing between a factitious disorder and a somatoform disorder.

**Case presentation:**

A 42-year-old Caucasian woman worked as a 100% fiduciary accountant until the age of 32 when she was placed on medical leave due to persistent trigeminal neuralgia. Afterward, she developed total blindness, not explained by a physiological process, accompanied by distress in a crucial emotional context. We evaluated the patient for a revision of a disability income after a diagnosis of factitious disorder with severe consequences such as disability income suspension and family conflict. Our psychiatric examination concluded the diagnoses of pain disorders related to psychological factors and a dissociative neurological symptom disorder with visual disturbance.

**Conclusions:**

Blindness not explained by a physiological process may accompany trauma and psychological distress. Differentiating this pathology from factitious disorder or simulation is essential from an insurance medicine point of view, but also for its treatment.

## Background

The disease burden due to non-specific, functional, and somatoform disorders is high [1]*.* A meta-analysis of 32 studies [2] investigated the prevalence of somatoform disorders [3, 4] and medically unexplained symptoms. A wide heterogeneity in prevalence rates was highlighted in primary care settings. The prevalence for the diagnosis of at least one somatoform disorder was 34.8% when only data from high-quality studies were included. The mean lifetime prevalence was 41%, and at least one medically unexplained symptom was diagnosed in 40–49% of all primary care patients [2]. A German study [5] shows that 91% of all patients with an F4 diagnosis, according to the 10th Revision of the International Classification of Diseases (ICD-10), are exclusively under the care of general practitioners (GP) and nonpsychiatric specialists.

An unknown part of these medically unexplained symptoms belongs to factitious disorders. It is challenging to estimate the prevalence of the factitious disorder in the general population. Due to the secretive nature of this disorder and the deception required to make a diagnosis, it is likely underdiagnosed [6]. The prevalence varies from 1.3 [7] to 6% [8]. The risk factors to present a factitious disorder include female sex, employment in the healthcare field, and being unmarried [9]. Most often, factitious disorder onsets in early adulthood or middle age [10].

Factitious disorder with physical symptoms is a psychiatric disorder in which sufferers intentionally fabricate illness, injury, or impairment to gain hospital admission and undergo medical procedures without any obvious gain [3]. When it comes to deciding whether or not a patient is able to work, from an expert point of view, it is essential to differentiate a somatoform disorder from a factitious disorder. The deliberate production of symptoms in the factitious disorder [11] is a reason to dismiss a disability income. Actual suffering and distress due to insufficiently identifiable symptoms and the desire for clinical improvement characterize the somatoform disorder. Patients suffering from a somatoform disorder could be eligible for a disability income when limitations related to their disorder are severe.

Through the case report that we present, we would like to throw some light, on the one hand, on the differences between somatoform disorder and factitious disorder and, on the other hand, the potential impact of misdiagnosis in medical expertise regarding eligibility for a disability income.

## Case presentation

A 42 years old Caucasian female, single, childless, had business school training, worked as a 100% fiduciary accountant until the age of 32, the date she was put on sick leave due to persistent trigeminal neuralgia. We investigated her in our multidisciplinary medical expertise service, in which evaluations from different medical specialties are conducted. In our service, a GP (IK) collects the information of all the specialists who participated in the assessment and discusses the case with the psychiatrist (BPM, KT) to pronounce himself regarding the working capacity review.

We evaluated the patient for a review of a disability income. When she was 32 years old, she had to stop her professional activity due to severe persistent trigeminal neuralgia. She made the first claim for disability benefits, which was agreed to 100%. At 38 years of age, the insured moved to another city, and the insurer requested a review of the income. Psychiatric expertise was conducted, in which a factitious disorder was retained as diagnosis, with a full working capacity. This diagnosis brought severe consequences such as a suspension of income due to disability and a family conflict that implied the patient was “inventing” the symptoms. It also impacted her symptomatology with an increase in stress and pain. She contested this decision, and our expert service received a reevaluation request.

The patient presented an essential left trigeminal neuralgia since 31, treated by glycerolization of the Gasser's ganglion, which temporarily improved her symptoms. Due to the recurrence of pain, and after a surgical intervention (left microvascular decompression), a radiosurgery treatment was performed on the trigeminal nerve, at its root, brainstem, and the left hippocampus levels. Three years later, a subcutaneous electrodes implantation treatment was performed without improving her symptoms. After this treatment, at the age of 36, the patient complained of visual disturbances. Progressive visual loss over 12 months lead to complete blindness by 39. At the age of 42, an extensive neuro-ophthalmological examination was performed (FXB). The patient reported a total absence of light perception in both eyes (subjective). The ocular examination revealed only the presence of severe punctuate superficial keratitis of the left cornea with left corneal anesthesia (normal sensitivity of the right cornea). Fundus examination revealed normal macula, peripheral retina, retinal vessels, and optic nerve in both eyes. Objective assessment of macula and optic nerve was normal by Optical Coherence Tomography (OCT) in both eyes.

Electrophysiological testing disclosed normal results for multifocal electroretinogram (ERG), full-field ERG, and pattern visual evoked potentials. The left neurotrophic keratitis resulted from previous therapies delivered to the left trigeminal nerve and is certainly responsible for some degree of visual impairment but could not explain total blindness of the left eye. There was no explanation for the blindness of the right eye. Electrophysiological investigations and OCT failed to reveal either any interocular asymmetry or any disturbances of the afferent visual pathways on both sides. Loss of vision was then attributed to a severe and chronic functional non-organic component superimposed on an organic left keratopathy resulting from the left trigeminal therapies.

During the psychiatric interview, we learned that her childhood was marked by the fear of losing her mother in the context of repeated hospitalizations. At the age of 7, she reported having been the victim of a sexual assault. When she asked for help from her father, he made her feel guilty. Regarding the aggressor’s identity, she described that she “can’t see his face". Later, her classmates mocked her for being the only one wearing glasses. When she turned 14, she was again sexually abused for almost a year by her schoolteacher. The sexual assaults took place in the classroom after school, the teacher pulling down the window blinds to get dark. At the age of 15, she got pregnant with twins. The perpetrator forced her to have an abortion in inhumane conditions while "blindfolded".

The patient started a Business School at the age of 15, describing that she was in "a denial of these traumas". She explains that defensively, to avoid thoughts linked with the traumatic experience, she heavily invested in her studies with good academic results. When she was 17, she came across the teacher who raped her, an encounter that provoked a traumatic experience's reactivation. It was in this context that she started psychotherapy. At the age of 30, she had her first boyfriend. She described that she decided to tell him that she was raped five months after the beginning of the relationship. He left her following this unveiling. After the end of this relationship, she described the onset of diffuse pains. She began to complain of abdominal and left ear pain with no improvement despite the proposed treatment, with an unfavorable evolution.

At 31, due to the painful symptoms on the left trigeminal territory, she benefited from two brain magnetic resonance imaging which were normal. A neurologist suspected left trigeminal neuralgia some months later, and an interventional analgesia treatment was performed. When she was 32 years old, she was admitted to a psychiatric hospital due to visual hallucinations, and a diagnosis of possible dissociative disorder was retained. In the same year, in the context of recurrent left trigeminal neuralgia, she benefited from a radiosurgery treatment with no improvement. Afterward, she underwent subcutaneous stimulation with electrodes at the infra and supraorbital level by the neurosurgeons, primarily without side effects. Due to the lack of improvement, neurosurgeons decided to remove the subcutaneous implants. Neurologists retained a functional nonorganic etiology, and they proposed a hypnosis treatment.

The visual disturbances started at the age of 36, during a psychotherapeutic treatment of exposure to trauma. The treating neurologist did not find any oculomotor alteration, and a thorough ophthalmological examination did not reveal any visual impairment or ocular abnormalities to explain the patient's complaints. They observed an apparent discrepancy between subjectively claimed visual loss and visual performance. A year later, a neuro-ophthalmologist (FXB) assessed her and found only left neurotrophic keratitis (secondary to iatrogenic trigeminal nerve injury), concluding to bilateral blindness of a non-organic functional type. At this time, the patient needed outside help to ensure her safety and independence.

In summary, detailed ophthalmological and neurological examination, electrophysiology testing, brain imaging, and blood tests revealed no significant abnormalities that could have contributed to his visual impairment.

At the age of 39, a psychiatric expert diagnosed a factitious disorder. From that moment on, different diagnoses of functional symptoms, factitious and somatoform disorders were proposed with no consensus. A deficiency of Vitamin B12 (99 pmol / l; norm 145–569 pmol / l) was observed and treated at the age of 41; Vitamin B12 level was normal during our examination (365 pmol / l). Sexually transmitted infections have been excluded during 10 years of follow-up in a primary care and specialized setting and there was no evident risk in order to test again in the context of the current expertise. On the date of our evaluation, she was under treatment of the following psychotropic drugs: clonazepam, alprazolam, vortioxetine and trazodone.

## Discussion

The patient presented with atypical left facial neuralgia since 32, initially attributed to essential trigeminal neuralgia. According to neurological assessment, after ten years of treatment-resistant evolution (including highly invasive treatments) and unremarkable investigations, we ruled out essential trigeminal neuralgia. We also ruled out neuropathic pain or a cluster headache. The neurologist confirmed an atypical facial pain, probably functional.

Except for left iatrogenic neurotrophic keratitis, the ocular examination was unremarkable, without any evidence of either diffuse retinopathy, maculopathy, or optic neuropathy. Functional non-organic blindness was diagnosed. Vitamin B12 deficiency can induce bilateral optic neuropathy [12], and a moderate deficiency was detected at 40. However, total blindness (no perception of light) was already present until the patient was 37 years old, without any evidence of optic atrophy. This deficit was efficiently treated with normal values in the follow-up control without any visual improvement. We concluded that the moderate vitamin B12 deficiency presented by our patient had no relationship with her reduced visual acuity (VA).

Alcohol consumption is related to visual impairment [13], and it is wrongly used in at-risk patients as an emotional anesthetic. However, the patient was asked about alcohol consumption during the interview, and the answer was negative. We also did not observe any symptoms of intoxication in our interviews. We reviewed her medical file, and there is no trace of a history of alcohol consumption and liver function test showed normal values.

Total blindness was not explained by a physiological process and was accompanied by distress in a crucial emotional context. A somatoform disorder in a patient with psychological suffering can explain the chronic pain. The sensory impairment in connection with the personal conception of the body’s function and the visual impairment can be explained by the conversion disorder with a sensory symptom or deficit disorder.

The thorough psychiatric examination (BPM, KT) concluded the diagnoses of pain disorders related to psychological factors, but above all, the diagnosis of a dissociative neurological symptom disorder with visual disturbance. A new diagnosis of dissociative neurological symptom disorder with visual disturbance (6B60.0) is included in the 11th Revision of the International Classification of Diseases (ICD-11). It is characterized by visual symptoms such as blindness, tunnel vision, diplopia, visual distortions, or hallucinations that are not consistent with a recognized disease of the nervous system, other mental, behavioral, or neurodevelopmental disorders, or other medical conditions and do not occur exclusively during another dissociative disorder [4]. This new definition more precisely characterized the patient’s symptomatology.

The criteria of a deficit and enduring personality change after a catastrophic experience were also clearly present. Following repeated traumas during her childhood, not having a sufficiently secure family setting nor stable attachment figures had not allowed her to build an identity base. Relationships with others were characterized by mistrust and fear, feelings of abandonment, or misunderstanding continuously emerging. Much of her energy was devoted to maintaining internal balance, struggling to avoid a depressive collapse, and containing emotional emergencies that could destabilize her. Fixations in somatic symptoms since the age of 32 had helped her control some of the internal tensions.

On the other hand, premeditated intentionality wasn’t observed; the patient sought recognition of her suffering, but she didn’t aim for a “sick status”. The insured never showed pain during the consultations, and there were no pain complaints during the session. We had enough elements to link the appearance of her symptoms with significant unconscious psychic conflicts and the revival of traumatic events. For example, the aggressor permanently closed the blinds and abused the patient in the dark, or the patient explained that when she had to undergo the abortion of her twins, they blindfolded her so that she could not see. We noticed a solid temporal link between the ophthalmic symptoms and the reactivation of traumatic experiences. Specifically, the ophthalmological symptoms arose after the separation of her only romantic relationship and her psychiatric treatment of exposure to trauma, in other words, around a “conflict context” with “identifiable stressors”.

There was no argument for a factitious disorder or a simulation, depressive reaction, or other psychiatric diagnoses (Table 1).

**Table 1:** Differential diagnosis and basic characteristics of the factitious disorders and the functional, dissociative, somatoform or bodily distress disorders [11]

	Description	Self-harm	Symptoms Production	Motivation and willingness to change	Objective findings	Comorbidity
Factitious disorder	Intentional production of symptoms to assume the sick role Can become life-threatening and take on the character of addiction	Significant; often requiring urgent medical intervention	Deliberate	Unconscious; external incentives are lacking or clearly in the background Low to ambivalent willingness to change	Abnormal, sometimes discrepant	Significant physical and psychological comorbidity
Functional, dissociative, somatoform or bodily distress disorders	Actual suffering and distress due to insufficiently identifiable symptoms Also present outside the examination situation Important areas of life are consistently impaired	None or mild	Not deliberate	Unconscious; external incentives are lacking or clearly in the background Predominantly high willingness to change	Mostly normal	Significant mental and possible physical comorbidity

The somatoform dissociation is present in different medical specialties under other terms. In the case of our patient, a non-organic visual loss (NOVL) was retained. Functional or NOVL is defined as a loss or decrease in VA or visual field range with no identifiable organic cause, however, non-organic blindness has been associated with specific neurophysiological correlates [14–16]. It is a condition found in both children and adults, with several reports citing an incidence of approximately 1.75% in children and 5.25% in adults presenting to the outpatient ophthalmology clinic [15]*.* The high prevalence of these disorders imposes a rapid and precise diagnosis to prevent intensive and unnecessary treatments and start an adapted treatment (psychiatric and psychotherapeutic treatment included) [16].

The relationship between somatoform dissociative pathology and trauma has been underlined. Dissociative disorders are characterized by loss of sensations and control of bodily movements and are often related to traumatic experiences like sexual abuse [17]. Childhood sexual trauma has been proposed as an essential source of somatoform dissociation [18]. Nijenhuis introduced the concept of somatoform dissociation, referring to dissociative symptoms that involve the body and comprise reduction up to complete loss of sensory perception [19, 20]. The concept of defense cascade can explain the relation between Post Traumatic Stress Disorder (PTSD) and dissociative disorders. Existential threat first prompts excessive psychological arousal; the lack of escape options turns into a “shutdown” response and can be described as a somatoform dissociation leading to functional neurological symptoms [21, 22]. On the other hand, a positive correlation has been observed between alexithymia, dissociative symptoms severity, and childhood abuse [23].

Regarding the visual hallucinations described by the patient, there are many research studies on the mechanisms linking childhood trauma and psychosis. The relationship between the two elements appears to be multifactorial. In fact, childhood adversities may interact with genetic vulnerability to psychosis and other environmental factors (substance abuse, low socioeconomic status or high urbanicity) [24]. This may lead to psychosis via distinct biological alterations such as: abnormal DNA methylation, hypothalamic–pituitary–adrenal axis dysregulation, decreased levels of brain-derived neurotrophic factor or subclinical pro-inflammatory in parallel to hippocampus and amygdala structural changes [25]. From a psychological point of view, childhood adversities might also increase a risk of psychosis through several psychological mechanisms, such as dysfunctional cognitive schemas, affective dysregulation, insecure attachment, and dissociation [25].

This case is also essential and unique because it shows the impact on the disability benefits and the subsequent psychosocial repercussions of misdiagnosis between factitious and somatoform disorder. A diagnostic error sometimes linked to poor management of the countertransference or misknowledge of somatoform dissociative pathology can have severe socio-economic repercussions with worsening somatic symptoms that can even lead to iatrogenic interventions.

The term "psychosomatic" is based on the humanist idea of the necessary link between psyche and soma. For this reason, the clinical approach of psychosomatic medicine is defined as "a comprehensive and holistic way of approaching the person"; in other words, a way of approaching the patient to tackle the disorder that leads to a medical consultation from the perspective of the whole person made up of body and psyche, linked and not just connected [26].

From a therapeutic point of view, meta-analytic evidence supports the efficacy of psychotherapeutic treatment for the somatic syndrome disorder and functional somatic syndromes [16, 27], early psychological interventions being promising but challenging [28]. Psychiatrists and primary care physicians should improve their ability to diagnose functional disorders and be comfortable treating this group of patients. Unnecessary referrals are frequent if the diagnosis is not felt to be accurate, and patients will be unlikely to accept the diagnosis and treatment plan [29]. On the contrary, when it is well explained within a solid doctor-patient relationship, and patient-involving treatments are proposed, chances of success are significantly increased [27, 30].

Psychotherapy should be explained in how it will help the patient’s symptoms. Participation in a treatment process that changes “the way the brain processes information” is essential to minimize the tendency to express distress through physical symptoms and create new behaviors that break the established, unconscious pattern that leads to those symptoms [29]. A positive diagnosis incorporating explanation and clinical assessment of visual ability seems benefic. Transparency in explaining how the signs work appears to help patients with functional disorders [31], patient-involving therapies being more effective than passive ones [30].

Other techniques such as transcranial magnetic stimulation or hypnosis have been tested. Mulckhuyse et al. [32] showed that occipital transcranial magnetic stimulation facilitates the production of visual perception in humans by producing light flashes called phosphenes [32].The production of phosphenes using this tool might help to demonstrate to patients their ability to have visual experiences and trigger better visual awareness [31]. Hypnotherapy, given after transcranial magnetic stimulation, may provide an additional benefit [31]. But, its mostly stepped care approaches, translating a biopsychosocial approach into patient management and concentrating on the doctor-patient relationship, saw the more solid results [27, 30].

## Conclusion

Blindness not explained by a physiological process may accompany trauma and psychological distress. Functional non-organic blindness is a challenging diagnosis and has been recently included in the 11th Revision of the International Classification of Diseases (ICD-11) as a dissociative neurological symptom disorder with visual disturbance (6B60.0). Differentiating this pathology from a factitious disorder or a simulation is essential from an insurance medicine point of view and for its treatment. Psychotherapy, biopsychosocial approaches, explanation of visual ability, and diagnostic transparency within a solid doctor-patient relationship are therapeutic.

## Data Availability

All data generated or analyzed during this study are included in this published article.

## Abbreviations

ICD-10: International Classification of Diseases 10th Revision
ICD-11: International Classification of Diseases 11th Revision
GP: General Practitioner
NOVL: Functional or non-organic visual loss
PTSD: Post Traumatic Stress Disorder
VA: Visual Acuity
